# Evaluation of Simple Lateral Flow Immunoassays for Detection of SARS-CoV-2 Neutralizing Antibodies

**DOI:** 10.3390/vaccines10030347

**Published:** 2022-02-23

**Authors:** Olaf Nickel, Alexandra Rockstroh, Stephan Borte, Johannes Wolf

**Affiliations:** 1 Department of Laboratory Medicine, Hospital St. Georg, 04129 Leipzig, Germany; olaf.nickel@sanktgeorg.de (O.N.); stephan.borte@sanktgeorg.de (S.B.); 2 Fraunhofer Institute for Cell Therapy and Immunology, 04103 Leipzig, Germany; alexandra.rockstroh@izi.fraunhofer.de; 3 Immuno Deficiency Center Leipzig, Jeffrey Modell Diagnostic and Research Center for Primary Immunodeficiency Diseases, Hospital St. Georg, 04129 Leipzig, Germany; 4 Division of Clinical Immunology and Transfusion Medicine, Department of Laboratory Medicine, Karolinska Institutet, Karolinska University Hospital Huddinge, 14152 Stockholm, Sweden

**Keywords:** COVID-19, SARS-CoV-2, vaccination, BNT162b2, neutralizing antibodies

## Abstract

Immunization for the generation of protective antibodies against severe acute respiratory syndrome coronavirus 2 (SARS-CoV-2) has emerged to be highly effective in preventing hospital admission, need for intensive care treatment and high mortality in the current SARS-CoV-2 pandemic. Lateral flow immune assays (LFIAs) offer a simple and competitive option to monitor antibody production after vaccination. Here, we compared the diagnostic performance of three different lateral flow assays in detecting nucleocapsid protein (NP), S1 subunit (S1) and receptor binding domain (pseudo)-neutralizing antibodies (nRBD) in sera of 107 health care workers prior (V1), two weeks (V2) after first vaccination with BNT162b2 as well as three weeks (V3) and eight months later (V4). In sera at V1, overall specificity was >99%. At V3, LFIAs showed sensitivities between 98.1 and 100%. The comparison of S1 and nRBD LFIA with S1 ELISA and a focus reduction neutralization assay (FRNT) revealed high concordance at V3. Thus, the use of lateral flow immunoassays appears to have reasonable application in the short-term follow-up after vaccination for SARS-CoV-2.

## 1. Introduction

The diagnosis of a SARS-CoV-2-driven infection and disease (COVID-19) is established by reverse transcription polymerase chain reaction (RT-PCR) of nasopharyngeal sample material, or detection of SARS-CoV-2 antibodies in the bloodstream. Following vaccination, the detection of SARS-CoV-2-neutralizing antibodies might guide clinical decision making in certain individuals, including but not limited to immuno-compromised hosts and -senescent patients. Although a correlate of protection is still missing, neutralizing antibodies (nAb) are estimated to be indicative for protection against severe courses of COVID-19 [1]. However, detection of nAb is time-consuming and harbors a number of technical pitfalls [2]. Therefore, simplified and point-of-care accessible assays such as lateral flow immunoassays (LFIAs) might represent a valuable alternative [3,4]. The performance of these tests can vary greatly and should be examined in detail before use [5,6]. We thus evaluated two different LFIAs in predicting the presence of neutralizing antibodies against SARS-CoV-2 Spike subunit 1 (S1) and receptor binding domain (nRBD). The spike protein is important to mediate the interaction between the virus and the angiotensin-converting enzyme-2 (ACE-2) receptor of the human host cell [7,8,9]. RBD as part of the spike subunit S1 plays a key role in the cell entry process [10]. This attribute explains that about 90 percent of SARS-CoV-2 nAbs in convalescent COVID-19 patients are directed against RBD epitopes [11]. The high proportion of RBD antibodies among the group of SARS-CoV-2 nAbs makes them a perfect surrogate marker for estimating the humoral neutralizing response in vaccinated individuals. For this reason, most commercially available LFIAs use RBD as a pseudo-nAb marker [12,13,14]. S protein and its receptor binding domain is the central antigen in all approved vaccines to induce protective antibodies. In contrast to S1 and RBD antibodies, nucleocapsid (NP) antibodies are only found in COVID-19 patients and in convalescent individuals. In this study, we analyzed venous blood samples of 107 healthcare workers from a German hospital with three commercially available LFIAs. The samples were collected at four visits following vaccination with BNT162b2 (BioNTech/Pfizer, Mainz, Germany). The aim of this study was to assess the performance of LFIAs in detecting NP, S1 and RBD antibodies in comparison with IgG anti-SARS-CoV-2 ELISAs and a SARS-CoV-2 focus reduction neutralization assay (SARS-CoV-2 FRNT). Based on the collected results, we would like to evaluate whether the LFIAs used are able to replace expensive laboratory-based antibody tests under certain conditions. In addition, it will be discussed how the tests used in this study rank in terms of their performance compared to competitor products. Furthermore, it is of interest how well lateral flow assays reflect the variations in antibody production over a long period of eight months after vaccination.

## 2. Materials and Methods

### 2.1. Study Population

Serum samples of 107 healthcare workers (median age 41 years (IQR 33–51), 70% females) from the St. Georg municipal hospital were included. All individuals received two doses of the BNT162b2 (BioNTech/Pfizer) vaccine according to national recommendations. Inclusion in the study was independent of a known previous SARS-CoV-2 infection. The ethics committee of the Saxonian medical chamber approved the study (registry number EK-allg-37/10–1). The samples were obtained prior to first vaccination (V1), on the day of the second vaccination (V2, median 21 days (IQR 21–21) after V1), 16–25 days after second vaccination (V3, median 42 days (IQR 42–43) after V1), and for 44 of 107 study participants 235–281 days after second vaccination (V4, median 253 days (IQR 276–282) after V1). Serum samples were stored at −20 °C in aliquots until further use.

### 2.2. Antibody Assays

Baseline antibody status and seroconversion after vaccination were monitored by commercially available LFIAs. A test for detection of IgG antibodies against S1 (LT-S1) and a surrogate virus nAb assay (LT-nRBD) were applied. For the latter, angiotensin-converting enzyme 2 (ACE-2) is precoated onto the test line region (T line) of the membrane. During testing, antibodies in the specimen (25 µL serum applied) react with RBD antigen, which is conjugated to colored particles. The mixture then migrates along the membrane chromatographically by capillary action and reacts with ACE-2 in the T region of the membrane. The presence of a colored T line that is more intense than the reference line (R line) indicates a negative result. A T line that is absent or weaker than the R line was assessed as a positive result. For detection of a possible natural infection prior or during the study period, an LFIA for assay of IgG antibodies against nucleocapsid (LT-NP) was performed in all samples. For LT-S1 and LT-NP, 10 µL of serum was applied to each. In contrast to the LT-nRBD, both tests were estimated as positive if a colored T line (weak to strong) occurred. All LFIAs were provided by nal von minden (Regensburg, Germany) and performed according to the manufacturer’s instructions.

For comparison, IgG antibodies against S1 (IgG anti-SARS-CoV-2 S1, Euroimmun, Lübeck, Germany; cut-off 25.6 BAU/mL) and IgG antibodies against nucleocapsid (NP) (IgG anti-SARS-CoV-2 NP, Virotech, Rüsselsheim, Germany; cut-off 9 VE/mL) were measured according to the manufacturer’s instructions.

In addition, a SARS-CoV-2 FRNT was performed using the strain BavPat1/2020. Heat-inactivated human serum samples were serially diluted 1:10 to 1:5120 and incubated with 50–100 focus-forming units of SARS-CoV-2 for 1 h at 37 °C before addition to confluent Vero E6 monolayers in 96-well plates. After an incubation of 1 h at 37 °C, supernatant was removed, cells were washed, overlaid with Methyl cellulose and incubated for 24–26 h at 37 °C in 5% CO_2_. Cells were then fixed with 4% paraformaldehyde, permeabilized and blocked with Perm-Wash buffer. SARS-CoV-2 focus-forming units were stained using a monoclonal human anti-S1 antibody CR3022 (abcam, Cambridge, Great Britain; 1:2000) and a secondary goat anti-human IgG HRP-conjugated antibody (Dianova, Hamburg, Germany). After the addition of TrueBlue substrate (Seracare, Milford, MA, USA), spots were counted with an ELISpot reader (AID Diagnostika, Strassberg, Germany). FRNT90 titers were determined for each replicate as the reciprocal of the last dilution, providing a minimum of 90% neutralization of focus-forming units in comparison to virus control without serum (FRNT_90%_). A titer of FRNT_90%_ ≥ 20 was defined as the cutoff for positive results. A detailed description of the procedure was previously published by Rockstroh et al. (2021) [15].

### 2.3. Statistical Analysis

Diagnostic sensitivity and specificity were reported with 95% Wilson confidence intervals (CI95%). Fleiss’ kappa was chosen as a measure of agreement. SPSS version 21 (IBM, Armonk, NY, USA) was used for statistical calculations.

## 3. Results

Analytical hallmarks of assay performance and concordance for SARS-CoV-2 antibody LFIAs and ELISAs are indicated in Table 1. For all assays under investigation, a specificity of more than 99% at V1 was observed. In addition, two individuals were correctly identified as true positives by all assays, supporting a known previous SARS-CoV-2 infection. One of the above joined V4 and was tested positive with the LT-NP, but negative by IgG anti-SARS-CoV-2 NP. In addition, another individual with a known vaccine breakthrough infection during the course of the study showed positive results with the NP assays at V4.

The assays IgG anti-SARS-CoV-2 S1 ELISA and LT-S1 displayed comparable sensitivities with high concordance from V2 to V4. The median values for the IgG anti-SARS-CoV-2 S1 ELISA at V2, V3, and V4 were 225, 3496, and 190 BAU/mL, respectively.

The sensitivity of SARS-CoV-2 FRNT and LT-nRBD increased from lower values at V2 towards the V3 time point. However, concordance with nAb at V2 was low (κ = 0.49) and considering V4, the LT-nRBD returned poor characteristics, whereas the SARS-CoV-2 FRNT displayed a sensitivity of 95.5% with a median titer value of 40. In this context, we performed a receiver operating characteristic curve (ROC) analysis by using the matched test results of SARS-CoV-2 FRNT and LT-nRBD between V2 and V4 to a sensitivity of >95% for the LT-nRBD. The ROC analyses revealed an AUC value of 0.873 (CI95% 0.831–0.914). Further, we determined a threshold titer of ≥80 for the SARS-CoV-2 FRNT to exceed 95% sensitivity in the LT-nRBD.

Two study participants who had already obtained their third vaccination with BNT162b2 presented with above average neutralizing antibody values (titer of 2560, respectively) applying the SARS-CoV-2 FRNT at V4. Both also showed positivity with the LT-nRBD.

## 4. Discussion

This study investigated the analytical performance of three different LFIAs in comparison to SARS-CoV-2 IgG ELISAs and a SARS-CoV-2 live virus neutralization assay. LFIAs are rapidly and easily performed even for medically untrained individuals in a home self-testing setting. This renders the possibility to conveniently and quickly assess SARS-CoV-2 immune status in larger populations without laboratory infrastructure, such as in countries with lower incomes and limited healthcare systems. NP-LFIAs could help to identify more naturally immunized individuals after unrecognized SARS-CoV-2 infections, which would prevent unnecessary vaccinations when vaccine supplies are limited. S1-LFIAs are suitable to monitor an adequate immune response after vaccination, especially for immune compromised persons. Positive nRBD lateral flow assay results could possibly be a surrogate for humoral protection against COVID-19 disease. With the help of these rapid tests, mass screening with LFIAs before major events would be conceivable. In contrast to the qualitative LFIAs, ELISAs allow precise quantitative results to be collected. In combination with standardized cutoff values, the method is suitable to monitor the kinetics of SARS-CoV-2 antibody production and determine the exact time corridor for a booster vaccination. With the help of the complex and more research-oriented FRNTs, such standardized cutoffs could be determined.

To our knowledge, this study describes the first relevant cohort size, frequency of sampling and number of LFIAs examined. In our hands, the LT-NP showed a specificity of more than 99%, which is comparable to the performance of other NP-LFIAs reported in recent studies [16,17]. Furthermore, these assays can discriminate individuals following SARS-CoV-2 infections in immune-naive conditions and after monotypic vaccination. Nevertheless, the number of subjects that experienced a SARS-CoV-2 infection within the study cohort was limited and the sensitivity of the LT-NP should be subject to further detailed investigations.

In contrast to other published reports, the herein studied LT-S1 assay revealed convincing results for analytical sensitivity and specificity (97–100%) [16,18]. The results of the LT-S1 corresponded well with IgG anti-SARS-CoV-2 S1 and clearly displayed a vaccine-induced S1-IgG immune response. However, qualitative LT-S1 are limited in their ability to assess the intensity of humoral immune responses after vaccination in comparison to a quantitative ELISA technique. This is especially so if one considers that the S1 subunit contains other domains beside RBD. This suggests that against these other protein structures, neutralizing antibodies are formed less frequently. However, an LT-S1 test concept could provide reliable additional information for the evaluation of COVID-19 patients with regard to eligibility and the usefulness of a monoclonal antibody (mAbs) therapy of acute SARS-CoV-2 infection [19].

The LT-nRBD presented with a lower sensitivity compared to SARS-CoV-2 FRNT. This explains the low concordance between the two methods two weeks after second vaccination (κ = 0.49). Nevertheless, at time point V3, the concordance between both assays was approximately 100% (κ = 1.0). In accordance with the results of Wang et al. eight months after the second vaccination, 75% of the individuals showed negative nRBD but detectable low nAb titers [20]. This indicates that the results of the nAb LFIAs are consistently relevant, as low nAb titers may be associated with higher rates of breakthrough infections [21]. However, nAb detected by the LT-nRBD were proved by a so-called surrogate virus neutralization assay. The main target of neutralizing antibodies against SARS-CoV-2 is the RBD. Previous studies have shown that anti-SARS-CoV-2 antibodies isolated from patients exert their antiviral activity via blocking the binding between the RBD and the target cell ACE2 [22,23]. Indeed, some antibodies can bind to the RBD epitope of SARS-CoV-2, which do not disrupt the RBD–ACE2 interaction. It is unclear if those antibodies affect the outcome of the LT-nRBD [22]. The performance of the two validated LFIAs of more than 95 percent sensitivity and specificity two weeks after second vaccination is equivalent to findings for other POCT in recent studies [4]. The two analyzed LFIAs are a valuable and reliable additional tool for SARS-CoV-2 nAb diagnostics, especially when no laboratory infrastructure is available. Compared to standard laboratory tests, the methodology has its weaknesses in terms of sensitivity over time after vaccination and in quantification of the results. A limitation of our study with potential impact on the practical application was the use of sera prepared from peripheral venous blood, whereas in a self-testing or outpatient setting, capillary blood would be of choice. Therefore, we suggest further investigation of these materials in subsequent studies.

## 5. Conclusions

In summary, our investigations gave new insights into the applicability and analytical relevance of lateral flow immunoassays for the detection of antibodies to SARS-CoV-2 following vaccination.

## Figures and Tables

**Table 1:** Test performances of three different lateral flow immunoassays (LFIAs) for detection of SARS-CoV-2 antibodies.

Test	Baseline (V1)	κ	V2	κ	V3	κ	V4 *	κ
IgG anti-SARS-CoV-2 NP		**0.65** (0.22–1.00)		**n/a**		**n/a**		**0.38** (0.00–0.91)
Sensitivity% (CI_95%_)	-		-		-		-	
Specificity% (CI_95%_)	**99.1** (94.1–99.9)		**n/a**		**n/a**		**97.6** (85.6–99.9)	
LT-NP								
Sensitivity% (CI_95%_)	-		-		-		-	
Specificity% (CI_95%_)	**99.1** (94.1–99.9)		**98.1** (92.6–99.7)		**98.1** (92.6–99.7)		**100** (89.6–100)	
IgG anti-SARS-CoV-2 S1		**0.79** (0.41–1.00)		**0.79** (0.41–1.00)		**1.00** (1.00–1.00)		**1.00** (1.00–1.00)
Sensitivity% (CI_95%_)	-		**98.1** (92.8–99.7)		**100** (95.6–100)		**100** (90.0–100)	
Specificity% (CI_95%_)	**100** (95.6–100)		-		-		-	
LT-S1								
Sensitivity% (CI_95%_)	-		**97.2** (91.4–99.3)		**100** (95.6–100)		**100** (90.0–100)	
Specificity% (CI_95%_)	**99.0** (94.1-99.9)		-		-		-	
SARS-CoV-2 FRNT (90%)		**1.00** (1.00–1.00)		**0.49** (0.33–0.66)		**1.00** (1.00–1.00)		**0.03** (0.00–0.17)
Sensitivity% (CI_95%_)	-		**56.1** (46.2–65.5)		**100** (95.6–100)		**95.5** (83.3–99.2)	
Specificity% (CI_95%_)	**100** (95.6–100)		-		-		-	
LT-nRBD								
Sensitivity% (CI_95%_)	-		**53.3** (43.4–62.9)		**100** (95.6–100)		**25.0** (13.7–40.6)	
Specificity% (CI_95%_)	**100** (95.6–100)		-		-		-	

LT-nRBD = LFIA for detection of (pseudo)-neutralizing antibodies, LT-NP = LFIA assay of IgG antibodies against nucleocapsid, LT-S1 = LFIA for detection of IgG antibodies against Spike subunit 1. * 44 of 107 study participants joined V4. n/a not available.

## Data Availability

The data presented in this study are available on request from the corresponding author.
